# Case Report: Laparoscopic Management of an Ectopic Pregnancy in a Rudimentary Non-communicating Uterine Horn

**DOI:** 10.3389/fsurg.2020.582954

**Published:** 2020-11-02

**Authors:** Kyriaki Chatziioannidou, Aurore Fehlmann, Jean Dubuisson

**Affiliations:** Department of Paediatrics, Gynaecology, and Obstetrics, Geneva University Hospitals and Faculty of Medicine, University of Geneva, Geneva, Switzerland

**Keywords:** ectopic pregnancy, mullerian anomaly, rudimentary horn, uterine rupture, case report

## Abstract

**Introduction:** Ectopic pregnancy in a non-communicating rudimentary uterine horn is a rare gynecological condition associated with a high risk of uterine rupture and important maternal mortality and morbidity. A surgical excision of the rudimentary horn is the standard treatment, usually performed by laparotomy in the second trimester.

**Methods:** A 36-year-old woman, secundigravida and nulliparous, was admitted to the emergency obstetric unit with acute pelvic pain. The ultrasound found an ectopic pregnancy at 15 weeks gestational age with fetal cardiac activity. As her hemodynamic status was stable, a diagnostic laparoscopy was performed and confirmed the development of the pregnancy in a left rudimentary uterine horn.

**Results:** We report a total laparoscopic removal of a pre-ruptured rudimentary uterine horn containing a second trimester ectopic pregnancy, using a vessel-sealer device. To our knowledge, only three other cases of successful laparoscopic treatment of second trimester rudimentary horn pregnancies have been reported in the literature.

**Conclusion:** Laparoscopy is an efficient and safe surgical option for treating rudimentary horn second trimester pregnancy in patients with hemodynamic stability.

## Introduction

The rudimentary horn is found in 0.5% of women (1). Pregnancy in a rudimentary horn is an extremely rare case of ectopic pregnancy. It presents a life-threatening condition due to a high risk of uterine rupture. In case of emergency it has been traditionally managed by laparotomy. Laparoscopic excision of ectopic pregnancy in a rudimentary uterine horn is also described as safe and successful during the first trimester in case of hemodynamic stability. We present the case of a total laparoscopic management in the second trimester.

## Case Description

A 36-year-old symptomatic woman, secundigravida and nulliparous, was admitted to the emergency obstetric unit at 15 weeks of gestation with a new onset acute pelvic pain.

The pregnancy was spontaneous with normal physical examination and harmonious evolution. The prenatal ultrasound of the first trimester has not suspected an ectopic pregnancy and the patient was totally asymptomatic until her admission to the emergency at 15 weeks of gestation.

When she was admitted to the emergency her hemodynamic status was stable. Physical exam revealed generalized abdominal tenderness dominating the left lower pelvic quadrant, no vaginal bleeding and a closed unique cervix, with no signs of a threatened miscarriage.

Transvaginal and transabdominal ultrasound showed an empty uterine cavity with no blood in the peritoneal cavity. There was a gestational sac with a viable embryo with a biparietal diameter of 35 mm. The gestational sac was next to the uterus, leading to diagnosis of a left tubal ectopic pregnancy of 15 weeks gestational age. As her hemodynamic status was stable, urgent diagnostic laparoscopy was decided.

Laparoscopy was performed using the Veress needle technique. The needle has been introduced in the palmer's point in the left upper abdomen to prevent any uterine injury. We have used one 5-mm supra-umbilical, one 5-mm suprapubic and two 5-mm ports in the left and right quadrants.

Both fallopian tubes and ovaries were normal, but a right hemi-uterus was found. The pregnancy was located in a left pre-ruptured rudimentary horn, surrounded by a 1 liter of hemoperitoneum. We associate the hemoperitoneum to the pre-rupture of the rudimentary horn prior to surgical management probably occurred at the meanwhile between the admission of the patient in the emergency unit and her transport to the operation room.

The surgery findings classified the Mullerian abnormality of the woman as V0C0U4a according to ESHRE classification (2).

**Figure 1:**
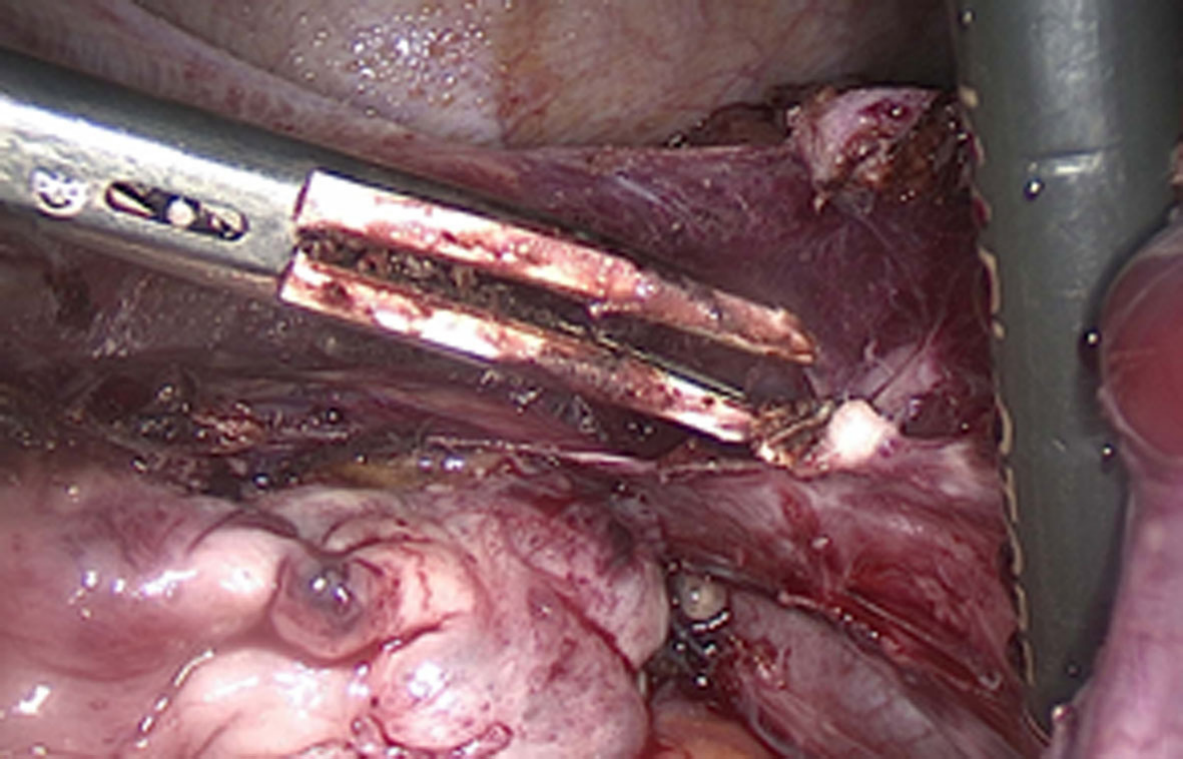
Left uterine artery.

No endometriosis was observed (Figure 1). We followed the surgical steps of a hemi-hysterectomy in order to remove the rudimentary horn and we also performed an ipsilateral salpingectomy. After identifying the attachment of the rudimentary horn to the hemi-uterus by a fibrous band stretched by the pregnancy, we performed the hemi-hysterectomy. The placenta was attached to this fibrous band.

**Figure 2:**
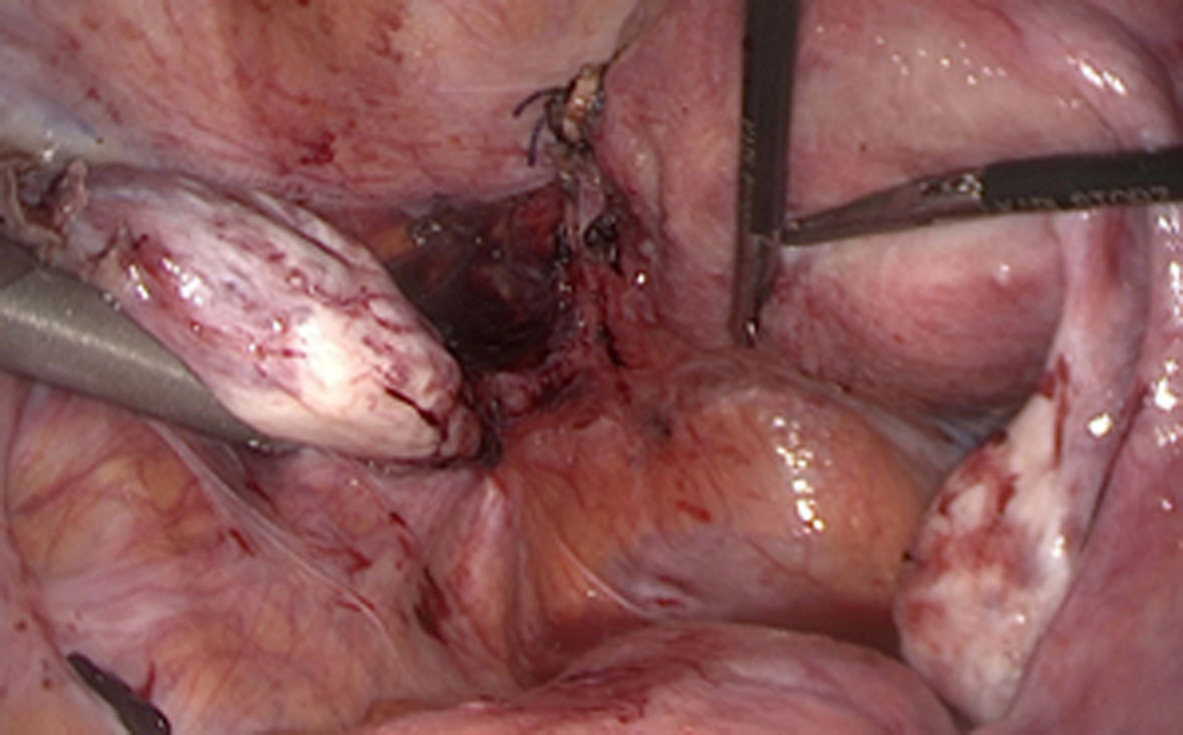
Final aspect after removal of the rudimentary horn with the pregnancy.

We performed a circumferential ligature with two tight knots around the attachment of the rudimentary horn to the hemi-uterus using Vicryl for absorbable suture. The separation between the two knots was completed using the LigaSure® device (Covidien, Medtronic, Minneapolis, MN). There was no bleeding during this separation. We should have performed a dye blue test in order to confirm no communication with the hemi-uterus but we suppose that there was no communication according to our perioperative findings. There was no defect in the myometrium of the hemi-uterus left in place after sectioning the attachment (Figure 2).

The rudimentary horn with the pregnancy was removed with an endoscopic bag through the previous abdominal incision extended to 3 cm in the left pelvic quadrant. With a gentle traction against the abdominal wall the fetus was removed intact. The rudimentary horn was extracted using mechanical morcellation under direct visualization into the endoscopic bag.

Blood loss estimation was 2 liters and the patient received a blood transfusion of two units, during the 180 min surgery, with no intra-operative complications.

The patient was discharged healthy on the first post-operative day. Follow-up was uneventful.

Histopathology confirmed a rudimentary horn of the uterus with a marginal hematoma.

No associated renal anomaly was diagnosed post-operatively.

## Discussion

Hemi-uterus is defined by the normal development of one unilateral müllerian duct with or without a rudimentary contralateral horn. In cases of partial development of the contralateral müllerian duct, the rudimentary horn is characterized by a functional or non-functional cavity. The contralateral part could also communicate or not with the hemi uterus (3).

The rare congenital abnormally of a hemi-uterus with rudimentary horn is usually detected during the investigations of infertility, recurrent abortions, endometriosis, hematometra, dysmenorrhea, preterm deliveries, intra-uterine growth restriction, and uterine rupture. Only 10% of these rudimentary horns communicate with the main uterine horn and 35% of them have a cavity which rarely includes a functional endometrium (3). One third of the cases report kidney abnormalities as well (4).

It has been described that rudimentary horn pregnancies are extremely rare and they are reported at 1:76,000–1:160,000 pregnancies (5). It seems that the ectopic pregnancy is occurred by transmigration of peritoneal sperm or fertilized ovum in the case of non-communicating uterine horn (6).

Early diagnosis of a pregnant rudimentary horn is challenging while the diagnosis is often missed on the prenatal ultrasound in the first trimester. It is reported that the sensitivity of the ultrasound is 26% and that it decreases with the advanced maternal age (7). The ultrasonographic criteria described for the diagnosis of a rudimentary horn pregnancy are the presence of an asymmetrical bicorporeal uterus, the absent continuity between the cervical canal and the lumen of the pregnant horn, as well as the presence of myometrial tissue around the gestational sac (8). In a second trimester pregnancy those criteria are often hardly identified.

The rate of uterine rupture in rudimentary horn pregnancies is almost 80% (9) associated to a 0.5% maternal mortality rate (10). As most cases are found out after uterine rupture, the early diagnosis of a rudimentary horn pregnancy is the most essential point to the successful management of this finding.

The surgical approach consists of the total excision of the symptomatic rudimentary horn and the removal of the ipsilateral fallopian tube in order to avoid the risk of a further ectopic tubal pregnancy (11).

The laparoscopic removal of the rudimentary uterine horn is described as successful with neither intraoperative nor postoperative complications (12). The first laparoscopic approach of a rudimentary uterine pregnancy was described in 1990 (13) and several case reports have been subsequently been published. To our knowledge, only three cases of laparoscopic management of a second trimester ectopic pregnancy in a rudimentary uterine horn are reported in the literature (14).

Knowing the two types of attachment of the rudimentary horn to the hemi uterus seems to be essential for the efficient surgical approach. The rudimentary horn is either broadly attached to the hemi uterus or minimally connected by a fibrous band. In each case the cleavage plane has to be well defined either at the beginning of the surgery or by radiological images before the surgery because the approach depends on the extension of the attachment of the rudimentary horn to the hemi uterus (3).

Suturing the myometrial defect can also be challenging to avoid a further uterine rupture. Good quality sutures are described as in “V” shape allowing to perfectly face the two edges of the myometrium (15). Modern conservative surgical approaches have been described in the management of ectopic pregnancies. Interstitial pregnancy was treated successfully with tubal curettage avoiding cornual resection (16). However, optimal suturing requires an experiment laparoscopic surgeon.

The management of a pregnancy in a rudimentary horn will also help us to establish a therapeutic strategy for the obstetrical future of the concerned patients. We recommend a c-section in case of childbirth, no pregnancy before 1 year and we propose an ultrasound follow up at 1 month and at 6 months postoperativly.

## Conclusion

Pregnancy in a rudimentary uterine horn is extremely rare and requires a precise surgical management. Laparoscopic approach of a second-trimester rudimentary horn pregnancy is a feasible and safe method.

## Data Availability Statement

The original contributions presented in the study are included in the article/supplementary material, further inquiries can be directed to the corresponding author/s.

## Ethics Statement

Written informed consent was obtained from the individual(s) for the publication of any potentially identifiable images or data included in this article.

## Author Contributions

KC and AF carried out manual search, draft of manuscript and revision according to other author's suggestions, and submission. JD carried out critically revision of the manuscript. All authors contributed to the article and approved the submitted version.

## Conflict of Interest

The authors declare that the research was conducted in the absence of any commercial or financial relationships that could be construed as a potential conflict of interest.
